# Registered nurses’ descriptions of caring: a phenomenographic interview study

**DOI:** 10.1186/s12912-015-0067-9

**Published:** 2015-03-28

**Authors:** Ewa Kazimiera Andersson, Ania Willman, Annica Sjöström-Strand, Gunilla Borglin

**Affiliations:** Department of Health, Blekinge Institute of Technology, SE-371 79 Karlskrona, Sweden; Department of Health Sciences, Lund University, SE-221 00 Lund, Sweden; Department of Care Science, Malmö University, SE-205 06 Malmö, Sweden; Department of Health Sciences, Karlstad University, Karlstad, SE-651 88 Sweden

**Keywords:** Caring, Conceptions, Interviews, Nursing, Registered nurses, Person-centredness, Phenomenography, Qualitative research

## Abstract

**Background:**

Nursing has come a long way since the days of Florence Nightingale and even though no consensus exists it would seem reasonable to assume that caring still remains the inner core, the essence of nursing. In the light of the societal, contextual and political changes that have taken place during the 21st century, it is important to explore whether these might have influenced the essence of nursing. The aim of this study was to describe registered nurses’ conceptions of caring.

**Methods:**

A qualitative design with a phenomenographic approach was used. The interviews with twenty-one nurses took place between March and May 2013 and the transcripts were analysed inspired by Marton and Booth’s description of phenomenography.

**Results:**

The analysis mirrored four qualitatively different ways of understanding caring from the nurses’ perspective: caring as person-centredness, caring as safeguarding the patient’s best interests, caring as nursing interventions and caring as contextually intertwined.

**Conclusion:**

The most comprehensive feature of the nurses’ collective understanding of caring was their recognition and acknowledgment of the person behind the patient, i.e. person-centredness. However, caring was described as being part of an intricate interplay in the care context, which has impacted on all the described conceptions of caring. Greater emphasis on the care context, i.e. the environment in which caring takes place, are warranted as this could mitigate the possibility that essential care is left unaddressed, thus contributing to better quality of care and safer patient care.

## Background

Nursing has come a long way since the 1860s and the ‘Nightingale Training School and Home for Nurses’ at St Thomas’s Hospital in London. Even so, it would appear reasonable to assume that nursing back then, as now, acknowledged caring as the inner core, i.e. the essence of nursing. Despite that caring has been regarded as the core of nursing for decades, our knowledge about caring still is mainly on a philosophical level. Numerous conceptualisations of caring have been presented over the years, using different theoretical and philosophical perspectives as the point of departure. During the 1940s and 1950s, there was a rise in the number of hospitals and hospital beds (cf. [1]) and a need for well-educated nurses. In that context Peplau [2] was the first to introduce, what was at the time, the revolutionary theoretical idea that nurses provide care to patients in a relationship based on communication. The theoretical development in nursing accelerated during the 1970s to 1990s, mainly in the U.S., but nursing theorists who focused on caring in nursing were few. In the 1970s, Watson [3] suggested that caring is the basic ontological substance of nursing and is interpersonal. Caring is a value and an attitude that manifests itself in the form of a concrete act. In a Scandinavian context, with a start in the 1980s, Eriksson [4] suggested that, in theory, caring, the innermost core of nursing, comes about in a relationship between the patient and the care provider and that caring implies alleviation of suffering. The problem with these philosophical descriptions is that they do not serve as explicit guides for today’s nurses, particularly in the light of the considerable changes that have taken place in the healthcare sector. In the 1990s, Morse and colleagues [5] described, in a concept analysis, five perspectives of caring: caring as a therapeutic intervention, an interpersonal interaction, a moral imperative, an effect and a human trait. Other concept analyses followed [6-8] and more recently Brilowski and Wendler [9] identified five core attributes of caring: relationship, action, attitude, acceptance and variability.

The concept of caring has also been studied empirically with qualitative designs [10,11], and it is explored in quantitative designs [12,13]. In a metasynthesis, Finfgeld-Connett [14] describes caring as a context-specific, interpersonal process signified, among other things, by intimate relationships and interpersonal sensitivity. Despite an *in absurdum* trail of research pursuing the quest ‘to say precisely what caring in nursing is’ (cf. [15] p. 188) the nursing community has yet to unite around a common conception of caring. Hitherto, caring has mainly been described, and explained using different semantics but with the same meaning – as ‘an act shaped in a relationship between nurse and patient’. However, in a recent editorial, Norman [16] puts forward the idea that the core of nursing might have evolved. He states: “Care and compassion continue to be prerequisites. But nursing has changed…” (p. 523). This makes it highly relevant to ask: Has caring, as the inner core of nursing, changed and, if so, what has changed?

Modern-day nursing, which takes place within a healthcare system that is undergoing wide-reaching reforms due to huge economic restrictions worldwide (cf. [17]), is under considerable pressure. Healthcare in the western world is facing significant challenges, for example changing patterns of disease, aging population, numerous people living with chronic illnesses and long-term treatments, and a mobile healthcare workforce [18]. Such transformations are followed by a focus on efficiency and productivity characterised by a very short length of stay in hospitals, increased care intensity, fewer hospital beds and a reduction in human resources, particularly registered nurses (referred to below as nurses) [19]. The main reasons for this development can be traced back to advances in medical science and technology, including information technology, as well as an increase in the cost of healthcare. There is also a shortage of nurses and a high turnover rate. As nurses are on the frontline of patient care, the suggestion is that the outcome of these developments put nursing under pressure, since nursing takes time to perform. There are alarming reports that both patient safety and patient well-being seem to have been lost [20] and that essential care is left undone [21-23]. At present, we do not really know if this is because caring within nursing have actually adapted negatively to changing circumstances or if this is the result of an increased workload and, in many places in Europe, a sub-standard level of education. The importance of having well-educated nurses (at undergraduate and post-graduate level) is supported in a study by Aiken et al. [19], which indicated that staffing hospital surgical wards with nurses with a degree and not making them care for more than six patients at a time, would lead to a significant reduction in preventable hospital deaths. It would be strange if nursing, and thus caring, were to remain in a vacuum and not be influenced by the pressure of 21st century economic, societal, political and contextual currents that demand large-scale change. Taking the above-mentioned developments into account it would appear realistic to ask what the core of contemporary nursing is, and how do nurses, as a group, perceive caring. The fact that this issue was under examination from the 1970s through to the 1990s makes these questions particularly pertinent. Scientific literature from this period contains descriptions of caring in nursing whereas contemporary literature, especially in the context of coronary care, is very sparse with regard to descriptions of caring in nursing and the few that do contain descriptions rarely do so from the perspectives of the nurses. Therefore, the aim of this study was to describe registered nurses’ conceptions of caring.

## Method

A qualitative design with a phenomenographic approach was chosen since the focus of this study was to describe the variation in how nurses could conceive, understand and conceptualise the phenomenon of caring [24,25]. Ontologically, phenomenography is not dualistic and assumes that the only world human beings can communicate about is the world that is experienced [25]. Epistemologically, the assumption of phenomenography is that humans differ in terms of how we experience the surrounding world, although the differences can be described, related to and understood by others. The conceptions may vary from one person to another, and within the same person as different aspects of the phenomenon are conceived depending on the entirety in relation to a given context. One strong point in phenomenography is that it offers a way of examining a collective human perspective of a phenomenon rather than individual perspectives. Phenomenography makes a distinction between first-order perspective, what something really is, i.e. the actual phenomenon, and second-order perspective, i.e. how something is conceived to be and how a phenomenon is conceptualised by people (ibid). In phenomenography, the second-order perspective is essential, which was adopted in this study.

### Context

For the past 15 years in Sweden, the only way into the nursing profession has been through a degree programme and since 2007 the only route is via a bachelor’s degree in nursing science, which involves three years’ study at university. Coronary care takes place mainly within secondary care (hospitals), where the nurses often work in a team with enrolled nurses (two years’ college education and no regulation) and a physician. The nurses can work in coronary care units (CCUs), thoracic care units or in thoracic intensive care units (which demands education in intensive care). This study was performed at two different county hospital CCUs in the southeast of Sweden. One of the CCU had access to round-the-clock invasive diagnostic, interventional therapy and cardiac surgery. The other CCU had no access to the aforementioned services, and the patients from this CCU were therefore taken by ambulance to the first CCU if diagnostic and/or interventional therapy was required. The two CCUs are divided into two parts – one emergency care part (six-seven single rooms) and one step-down/rehabilitation part (6–11 rooms with 16–19 beds) with telemetry facilities.

### Recruitment and participants

The nurses were recruited with help from one contact nurse on each CCU, who handed out an information letter to in total 23 of 70 eligible nurses. A purposive sampling strategy [26] was used to ensure varied and rich information through a heterogeneous sample in regards to age, education and length and variety of working experience (ibid). Two nurses declined to participate because of heavy workload and a need to work overtime. The final group of participants comprised of 21 nurses, 20 women and one man. Their ages ranged from 23 to 63 years (median = 46.0 mean = 44.1), 16 had a diploma, five had a Bachelor of Science in Nursing and five had a specialist education (n = 1 intensive care; n = 1 cardiac care, acquired outside Sweden; n = 3 medical and surgical care). Their overall working experience ranged from one to 42 years (median =20.0 mean = 19.0) and in the CCU from 0.5 to 27 years (median =16.1 mean = 18.0). Seven of the nurses shifted between the emergency and step down/rehabilitation part of the CCU, one worked only with secondary prevention and cardiac rehabilitation and the remaining 13 shifted between both parts of the CCU and nurse-led follow-up outpatient clinics (heart failure, arrhythmia and anticoagulation therapy e.g. warfarin) or cardiac rehabilitation. Eleven participants worked full-time and ten worked part-time.

### Data collection

The first author conducted the interviews between March and May 2013. All interviews began with a single, open-ended question: ‘Please tell me what caring means to you in your clinical work as a nurse?’ To check the suitability of the question it was tested among nurses with and without experiences from coronary care. It was also tested in a pilot interview, which was carefully scrutinised by the last and the first author in order to assure the latter’s interview technique. Follow-up prompts focusing on ‘how’ and ‘Can you tell me more’ were used to elicit and clarify. The interviewer was familiar with the care context (i.e. as a cardiac nurse). This can be regarded as important in order to pose relevant follow-up prompts based on the immediate understanding of what the participants were trying to express. The majority of the interviews took place in an undisturbed room at the participant’s workplace although two were conducted at the participant’s home. The interviews lasted from 31 to 59 minutes and were audiotaped and transcribed verbatim.

### Data analysis

**Figure 1:**
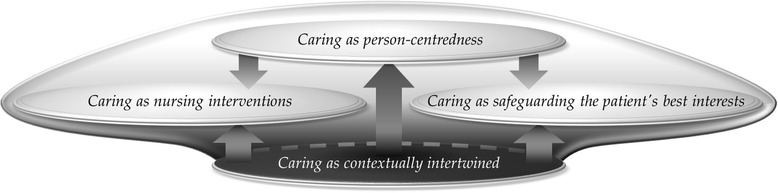
**The outcome space - Nurses’ collective understanding of caring.**

The analysis was conducted in a stepwise manner, inspired by Marton and Booth’s [25] description of the method since their description is a rich source for this method. In the first step, using an inductive approach, the interviews were read and re-read to gain an overall impression of the data. The parts of the participants’ statements were then identified in accordance with the aim of the study. These statements were then re-read, condensed and summarised as preliminary ways of understanding the phenomenon. In the second step, the condensed and summarised statements were carefully compared in order to identify variation and then similar statements were grouped or classified into preliminary descriptive categories. In the third step, through careful scrutiny, a comparison of the preliminary descriptive categories was made to establish the borders between them. Finally, through interaction between the whole and the parts, based on similarities and differences, four descriptive categories emerged. An iterative process was used throughout the data analysis to check interpretations against the texts and the descriptive categories. Hence, a continual process of iteration between a focus on the whole and the parts was used to check on the interpretation until the descriptive categories that emerged from the data were found to be mutually exclusive. The descriptive categories were used to form the ‘outcome space’ (Figure 1), in accordance to Marton and Booth [25] aiming to mirror the internal relationship between the four qualitatively different descriptive categories, hence representing the whole of the findings.

### Ethics

The study was performed in compliance with the ethical guidelines of the Declaration of Helsinki and was approved by the Regional Ethical Review Board in Lund (H15 2010/101 and 2012/406). To ensure confidentiality, each quotation was assigned a pseudonym in the form of a capital letter and number.

## Results

Our results were interpreted as reflecting four descriptive categories: caring as person-centredness, caring as safeguarding the patient’s best interests, caring as nursing interventions and caring as contextually intertwined, which reflected the nurses’ conceptions of caring.

### Caring as person-centredness

In the descriptive category *caring as person-centredness*, the nurses conceived caring as creating a genuine patient-nurse encounter in which the nurses use the holistic perspective as a point of departure. Caring was conceived as *‘seeing through’* the patient, i.e. the person behind the patient. The nurses described themselves as a tool for caring, and their ethical stance was considered to be crucial to the enhancement of caring. They strived to explore and reach an understanding of the person’s individual illness narration and the impact of the illness on everyday life and to do so with sensitivity and based on the person’s overall situation. This understanding was then used when caring for the patient. The nurses expressed *‘a sincere desire to listen and to help’* based on a *‘love of their fellow human being’* and it was stated that caring made the person the core of attention. The nurses stated that courage and inner strength were needed to receive and confirm each person’s narration. It was also considered important to be open, perceptive, reflective and creative when formulating individual caring solutions. One participant stated:

To see the whole patient in his/her context, how it (the illness) affects their life, when it comes to work, leisure, family life and such, for several years to come. (pause) It’s really important that you listen to what they’re wondering about, how they feel it has affected their situation and family and listen out for what it is they want to know… that you are sensitive to and really find out how it has affected this particular person and not generalise. This is how it usually is but really listens to how it is (pause). Try to ask open questions to get more comprehensive answers. (B6)

The nurses conceived caring as maintaining and strengthening the patient’s sense of dignity and being a person. The nurses stated that this meant being truly present, readily available and conveying a sense of comfort. The person’s experiences, feelings and preferences were considered to be vital aspects of caring. Showing respect for the person’s decisions or opinions, especially when they were not in accordance with the opinion of the professionals, was regarded as being essential to caring. The nurses stated that the patient was *‘the expert on her/his body and illness’* and emphasised the importance of involving the next of kin in caring. Being emphatic, compassionate and having knowledge of human nature were also conceived as caring. One participant stated:

Then you need that knowledge in order to (pause) what can you call that kind of knowledge? (pause) In a way it’s knowledge of people (pause) knowledge of human nature, that you’re able to read another person and dare to (pause) be open to what they say and dare to accept that. … Am I ready to hear what the patient is saying? Because it can be something that affects me that becomes difficult, something that is close to me personally or just from a life point of view, sort of existentially difficult… you need to have been working with these sorts of questions yourself and given it some thought and reflection … but it must be terribly hard if I worry about something. It must be hard to listen to a patient expressing concern about this, since this will stir these feelings in me, instead of being able to listen to what they’re saying. (A5)

### Caring as safeguarding the patient’s best interests

In the descriptive category *caring as safeguarding the patient’s best interests*, the nurses conceived caring as carefully guiding the patient through teamwork with other allied healthcare professionals. The nurses stated that they are bearers of important information and knowledge about the patient’s present situation and desires, thus acting as intermediaries between the patient and the other team members. Questioning decisions made without including the patient’s opinion was described as *‘standing by’* the patient. One participant stated:

I think that it’s important that I as a nurse (pause) Well, sometimes the decision is made before the physician has seen the patients and I think: Hey, this is a person we’re talking about, have you seen her? Have you heard her whole story? A bit of defending and presenting, personalising… We need to know what kind of patient we have in front of us and that means ethics. And it’s not easy; it’s not easy but you have to discuss it. (pause) … Then you also have to remind the physician properly, which I usually do. I’ve been doing that quite a lot lately during rounds. Did you look her in the eye? Did you make sure she understood what you were saying? (A9)

Caring was also conceived as protecting patients from unnecessary suffering. Caring could thus be said to be questioning whether prescribed examinations and tests, especially in conjunction with palliative care, were actually in the best interests of the patient. Documenting the care process, including the individual patient’s care plan, in the patient’s records was also stated to be a caring action aimed at promoting patient safety and the patient’s best interests.

Caring as safeguarding the patient’s best interests was described as becoming involved at an early stage in the patient’s discharge plans. The nurses described caring as early evaluation of the patient’s social situation, needs and desire for additional care following discharge but also as clear communication and a pathway between the hospital and municipal homecare. Limiting the number of healthcare professionals involved in follow-up was said to contribute to the continuity of care and caring, safeguarding the interests of the patient. Showing respect for the patient’s integrity, respecting the physical and psychological features of the patient’s privacy and accepting the extent to which the patient wanted to participate in their own care, were all regarded as being a part of caring that was just as important as safeguarding the patient’s interests. One participant stated:

Taking care of people on their own conditions, to ask them how they usually do it? What do you prefer when showering, how do you want to? That you ensure that it’s done on their… I can feel that it’s a form of mistreatment not to do as they, as the patient wants it. (A4)

### Caring as nursing interventions

In the descriptive category caring as nursing interventions, the nurses conceived caring to be vigilant patient surveillance resulting in care activities that lead to either relief or improvement of symptoms and enhanced well-being. Assessing and observing vital signs and physiological readings, while at the same time striving to understand the patient’s symptoms and body language, were stated to be an example of an intervention in caring. Integrating vital patient data, both objective and subjective, acting in complex situations, making decisions and knowing whether to act or to remain in watchful waiting are also conceived to be caring interventions. One participant stated:

It’s important that you know the technical aspect so that we can observe and see that: No this is pretty unstable, so you can sound the alarm and maybe change your mind and initiate various measures earlier than you possibly planned. … At the same time you look at them (the patient) and you then see your patient and watch the patient as well. How is the patient doing now? Any more pain? Completely unaffected? Sleeping? So that you really observe how it is compared to the other. … It’s also very important that you know those bits and that you observe it, observe the equipment, what the patient says and how the patient is feeling, the patient’s overall condition. … It’s also about (pause) letting the human being come out (pause) and making them part of the entire caring process. It’s really important that we get them on board.. ... Help the patient feel well physically and mentally. (A7)

Caring was described as collecting data about the patient to form a basis for diagnosis, assessment and action in accordance with the patient’s needs, e.g. pain. Caring was also conceived as pharmacological intervention, i.e. drugs, and non-pharmacological actions, such as *‘touching’* and *‘changing the patient’s position’* to relieve symptoms. Assuming responsibility for promoting health by administrating and evaluating the effects of different types of treatment was described as other nursing interventions, as were risk identification and assessments aimed at preventing falls, ulcers and/or malnutrition, and taking action to manage situations in collaboration with the patient. Assessment of the essentials of care, i.e. keeping patients clean, fed, hydrated, calm and involved, was also conceived as being part of caring as nursing interventions. One participant stated:

I think caring involves helping a seriously ill patient who can’t wash himself to be washed and be taken care of and have food. A seriously ill patient (pause) the main thing is that they’re clean, smell good that they get some food or liquid and if they can’t take it I think it’s important that they receive food in some other way. So it’s the seriously ill patients that I help with everything if that should be the case (pause) make conversation when I’m in there changing diapers but for me it’s important that they’re clean and not in any pain. (B3)

The nurses described caring as nursing interventions as continuously mediating customised information about treatment, medication and the current situation to and between the patient and the next of kin. It was considered important to situational caring and to the patient’s capability, situation and needs. One participant stated:

I think it’s important to start from the patient, where he/she is coming from and what level that is. The patient will start talking with me about how it’s been, explain their situation and when you’ve listened to the patient talk, that’s when you get to talk… it’s important that you let the patient be in control, that it’s he or she who knows their illness and their condition the best, so that they can tell you what it’s really like… you listen actively to what the patient has to say and then interpose with information during the conversation; it can be like that, it’s common that it’s like that, during the conversation… it is the patient who is (pause) the specialist regarding his body and his illness and then I need to listen actively and provide information based on this. (B2)

It was stated that using the patient’s perspective as a point of departure facilitated the intervention and made it easier to take in and understand communication regardless of native language and cultural customs.

### Caring as contextually intertwined

In the descriptive category caring as contextually intertwined, the nurses described how caring is intertwined with the context in which caring takes place. Hence, a heavy workload and inadequate and insufficient staffing, coupled with limited face-to-face patient time, were stated to be intricate features of caring. Caring as contextually intertwined was also described as prioritising measurable medical tasks and essential patient care while retaining composure in encounters with patients and when needing to leave essentials of care unaddressed. One participant stated:

… lack of staff. If there’s a lot to do, as always, (pause) it might actually be the case that caring is neglected sometimes, because then it’s just what’s most urgent that you prioritise all the time and unfortunately a lack of staff is a hindrance. There isn’t the time because then you constantly prioritise what’s most important and then even important essentials of caring are neglected … someone might be dying, for instance. It’s always the critical things, people who are very seriously ill. Those are the sorts of things that are prioritised all the time. (pause) And then other essential parts of caring are neglected because of that. And it might just be that there simply isn’t enough time and you wish there were more staff. (B8)

Caring in a context is in a constant state of evolution and is stated to be a challenge to the nurses’ theoretical and practical knowledge. Caring as contextually intertwined was therefore described, as being solely responsible for acquiring the clinical competencies and knowledge needed to adequately address care needs. One participant stated:

It’s important that you, that you’re well informed so you know what you’re doing and that you constantly keep up with all the new things that are going on so you can answer all the questions because a lot of patients today are really well informed… it’s really up to us that we look up the information ourselves and read, for example, the cardiology journal and read up on new studies, about new drugs and advice and findings, that you get to go away on conferences once in a while; it’s really important to be given a little push so to speak and pull yourself together because that makes you instantly more motivated. … now it is up me to look up the information and read. (A7)

However, it was also stated that important enablers of caring were cooperation with other nurses in charge, encouragement as well as reassurance from colleagues and managers and these were described by the nurses as important features of caring as contextually intertwined.

### The outcome space

Our results reflect a hierarchical relationship between the four descriptive categories, the outcome space (Figure 1), interpreted to represent the nurses’ collective understanding of caring.

The descriptive category caring as person-centredness reflected the most comprehensive way in which the nurses understood caring, i.e. the core of caring. This category was interpreted to reflect the nurses’ endeavour to find their way through to the person behind the label ‘patient’. The goal of their endeavour was to acquire an understanding of how the person experienced their illness and to base caring on this understanding. This stood out as being the core of their conception of caring and the main supporting feature of caring as safeguarding the patient’s best interests and caring as nursing interventions. In the latter, the nurses independently initiated caring that was aimed at relieving or improving symptoms and enhancing well-being. This, together with always putting the patient first, stood out as the main constituent in caring as nursing interventions. Carefully guiding the patient and acting as an intermediary in the patient’s best interests through teamwork with other allied healthcare professionals and ‘standing by’ the patient, are described as caring as safeguarding the patient’s best interests. Caring as contextually intertwined was described as surrounding the other three descriptive categories, even if it has the lowest hierarchical level in the outcome space. This indicates that caring was not perceived as a passive intervention occurring in isolation but rather as a dynamic contextual intervention occurring in intricate interplay in the relationship with patients, colleagues and the context in which caring takes place (Figure 1).

## Discussion

Our findings imply that nurses’ conceptions of caring have evolved during the last decade. The nurses in our study conceived that the starting point for caring is the perspective of the person behind the label ‘patient’ and their descriptions mirrored the intention to reinforce the patient, i.e. person-centred caring. By allowing the patients to speak about her/his illness and making the narration into a starting point for establishing care, nurses encouraged them to actively participate in health decisions. It would seem reasonable to suggest that this perspective emanated from the current all-embracing hierarchical systems at the hospitals [27,28], which also encompass nursing. A paternalistic, and at times maternalistic, tradition coloured the healthcare system, and it was a place where physicians knew better than nurses and nurses knew better than patients. One explanation for this change of focus in caring could be the strong democratic movements during the 21st century [29] where patients are now seen as active participants in healthcare decisions [30]. Another explanation could be published reports and studies that indicate that the healthcare system is failing to offer safe, good-quality healthcare [20-22]. It would appear important to note that our nurses’ novel description of caring as person-centredness cannot be attributed purely to changes in professional values, particularly as our findings also reflect a situation where the description of caring seem to be strongly linked to context. The move towards person-centredness most likely comes down to “….changes in the dominant views of society and lack of confidence in healthcare professionals, not simply because the healthcare professionals have adapted their thinking to be more respectful of patients’ rights” ([27] p. 696). This study showed that nurses conceive caring as person-centredness, which can be regarded as a good prerequisite for implementing person-centred care (PCC) in hospital settings. Ekman et al. [31] provided constructive routines to facilitate and safeguard the transition to PCC and to ensure that it can be practised systematically and consistently. Studies evaluating the effects of PCC interventions in Swedish hospital settings produced promising results [32,33], which imply that a paternalistic tradition no longer has a place in modern healthcare.

Caring stood out as being an act that takes place in collaboration with the patient while focusing on the patient’s best interests. The nurses described, how as a collaborative unit, they stood by the patient and where the many faces of caring were described, i.e. guiding, protecting, supporting and respecting. Caring was also described as giving the patient freedom of choice with regard to when to take on the role of patient and when to take on the role of a person. Others support this description and Karlsson et al. [34] suggest that the act of ‘standing by’ the patient is of importance and means that the nurse is attentive, present and prepared to assist the patient in all situations, whilst at the same time giving the patient the freedom of choice to decide the extent to which they want to participate. Furthermore, the nurses conceived caring as acting as an intermediary and working in a team. This implied an awareness of the importance of possessing the collaborative competencies needed in today’s healthcare organisations, which are often accused of offering ‘discontinuity of care’. We know from other areas [35] that caring described in this way can result in higher survival rates, shorter admission times and better potential for patients to regain independence. Teamwork is also known to improve patient planning, is clinically more efficient and supports a person-centred care [36]. Collaborative care seems to be of vital importance in times characterised by an intensified workload and strained healthcare finances, often resulting in inadequate nursing staff (cf. [20]). Working in multidisciplinary teams in order to ensure continuity of care, patient safety and quality of care is also one of the six core competencies suggested for nurses to possess in order to meet healthcare standards [16,37].

The more recently acknowledged complexity of nursing [16,38] demands that caring nowadays, more than in the past, is based on sound clinical judgements and an understanding of the concept of nursing, i.e. the idea that something is done for the patient is a thing of the past. Our findings thus reflected caring as interventions taking place with the patient and not for the patient. The results show that nurses conceived caring as undertaking nursing interventions, hence caring aimed at improving the patient’s symptoms, enhancing their well-being, promoting health and meeting the patient’s need for essential care and information while at the same time consistently putting the patient’s interests first. A nursing intervention is defined in the literature as ‘any treatment, based on clinical judgement and knowledge, that a nurse performs to enhance patient or client outcomes’ ([39] p. 2). However, it is important that nurses demonstrate interpersonal sensitivity and are attentive to the patient’s values, goals and preferences when undertaking nursing interventions. Inattentiveness and insensitivity to the patient’s specific needs in emergency nursing were found to be instrumental behaviour, i.e. uncaring [40] and routine nursing interventions as standard packages have been experienced by patients and their next of kin to be stressful and demanding [41,42]. The principles behind any intervention that nurses engage in must achieve positive nursing care outcomes, which according to Cronenwett et al. [37], requires nurses to have the ability to integrate current evidence with clinical expertise and the patient’s preferences and values. This also implies constantly questioning the rationale for routine care. As nursing care in some countries is nowadays subject to intense scrutiny, criticism and demands for change [20], the requirement for nursing care to be founded on solid evidence and on high-quality interventions is more pressing than ever. According to Conn [43], it is known that a large gap exists between daily nursing practice and available empirical evidence regarding interventions. Nurses therefore need to integrate evidence into their daily nursing practice in order to ensure research can be used for the benefit of all patients.

Our analysis clearly suggests that caring needs constant upkeep and a conductive environment. Caring is not a passive, static set of interventions occurring in isolation and without a strong connection to the context in which it occurs. In simple terms, caring demands time and knowledge. In our study the nurses described how they found themselves forced on a daily basis to focus on measurable medical tasks and to learn how to control their frustration when they were required to leave the essentials of care unaddressed. There is substantial evidence in the literature of an association between contextual aspects of caring, e.g. staffing and patient outcome. In a systematic review, Kane et al. [44] found that increased staffing levels were associated with lower odds of hospital-related mortality and adverse patient events. Similar results are shown in several recent studies across the globe [45-47]. Aiken et al. [19] showed a relationship between a decrease in inpatient hospital deaths on surgical wards staffed by nurses with a degree. Furthermore, and as shown in this study, caring that takes place in contexts that are undergoing substantial changes demands that nurses have the requisite competence and up-to-date knowledge, especially since healthcare can be regarded as a knowledge-intensive sector. Nurses’ decision-making and problem-solving in caring need to be founded on solid evidence as only then, according to Cronenwett et al. [37] can the nurse determine when to deviate from evidence-based practice in order to meet a patient’s preferences and values and deliver high-quality PCC. Unfortunately, in another study by Aiken et al. [23] it was indicated that one in five nurses across Europe were dissatisfied with their jobs due to uncertain opportunities for education and advancement. It therefore seems reasonable that continuous in-service training should be a natural part of the nurse’s day-to-day work. This demands a sustained organisational structure that supports development and utilisation of research in daily practice, consistent clinical supervision and, according to Norman [16], a nursing degree. Our interpretation is that greater emphasis on the care context, i.e. the environment in which caring takes place, is warranted as this can minimise the possibility of essential care being left unaddressed and thus contribute to better quality of care and safer patient care.

### Methodological limitations

Twenty-one purposefully sampled nurses, varying in age, education and work experience and from two county hospitals CCUs, were included in this study, which can be regarded as sufficient to ensure variation in ways of perceiving and conceptualising the same phenomena [25]. The two county hospitals are located in a sparsely populated area, which could influence the transferability of the findings due to possible contextual differences compared with for example a university hospital located in a larger city. However, it is worth noting, that one of the two hospital CCUs, that the participants were recruited from, provides a level of service that is fully comparable with any university hospital, i.e. the CCU had access to round-the-clock invasive diagnostic, interventional therapy and cardiac surgery. Although the sampling was purposively conducted, it was uniform with regard to gender (majority was female and only one male). Nevertheless, it was considered representative of the nursing profession, since, in Sweden, only approximately 11% of nurses are male. This, together with the informants’ heterogeneous education and work experience, might increase the transferability of our findings to similar contexts. An evaluation of the trustworthiness of this study could be performed within the framework of its credibility [48]. To accomplish this and enable a transparency of analysis, direct quotes from the interviews are presented giving the reader the opportunity to judge the interpretation as well as its relevance to similar settings (cf. [49]). To reduce the risk of subjectivity [50], the authors regularly worked together throughout the analysis to strengthen it, not by achieving consensus or arriving at identical formulations in interpretations, but by supplementing and contesting each other’s readings. Only one of the authors (EKA) had any experience of the participants’ setting, but the cooperation ensured that no preconceptions interfered with the question developed or the analysis. On the other hand, the limited exposure might also have caused us to miss some aspect of importance. Some of our descriptive categories could be said to reflect partially ‘idealised’ descriptions of caring. However, just like normative philosophical descriptions of caring, mirroring the content of nursing education, these descriptions are not strange when considering the relative lack of empirical studies investigating how contemporary caring can be understood. Although this study highlights some valuable learning points, it is important to note that it is a study performed in a specific setting and context. These characteristics should be taken into account when evaluating the study and its results.

## Conclusion

Our findings imply that the diverse conceptions of caring described by nurses could lead to the nurses adopting different focuses and actions. By acknowledging caring as person-centredness, caring as safeguarding the patient’s best interests, caring as nursing interventions and caring as contextually intertwined, caring could open up the possibility of influencing the patient’s experiences and well-being in different ways. Keeping track of how caring evolves and nurses’ current conceptions of caring would seem to be more important than ever. We propose that on a universal level, caring, regardless of context, is coloured by humanism and the most important prerequisite for caring, is to recognise and acknowledge the person behind the patient. In line with this, Drummond [51] explores the theory of avant-garde and nursing and states that nursing, despite the fact that the western world is going through profound changes, must always return to its basic principles, which are its human condition (humanitus). Drummond suggests that progress or improvement should always involve returning to something and thinking of something in a new way, i.e. reconnaissance. Regardless, and perhaps somewhat controversially, it could be the right time to suggest that it is no longer sufficient to explain caring as a unique nurse-patient relationship. Our results imply that caring is strongly linked to the context. Although we know that caring occurs in a complex healthcare environment, caring must be enacted in such a way that it is possible to observe, measure and replicate successful caring to the benefit of all patients. It is important to note that the focus of this study was on describing the nurses’ different conceptions of caring and not the concept of caring itself. Using an ethnographic approach, observations of how nurses provide care in practice would be an interesting subject for future studies.

## Abbreviations

CCU: Coronary care unit
PCC: Person-centred care
